# An NF-κB/OVOL2 circuit regulates glucose import and cell survival in non-small cell lung cancer

**DOI:** 10.1186/s12964-022-00845-z

**Published:** 2022-03-28

**Authors:** Rui Zhang, Guo-Jun Geng, Jian-Guang Guo, Yan-Jun Mi, Xiao-Lei Zhu, Ning Li, Hong-Ming Liu, Jun-Feng Lin, Jian-Weng Wang, Guang Zhao, Guan-Zhi Ye, Bo-An Li, Qi-Cong Luo, Jie Jiang

**Affiliations:** 1 Department of Thoracic Surgery and Xiamen Cell Therapy Research Center, The First Affiliated Hospital of Xiamen University, School of Medicine, Xiamen University, 55 Zhenhai Road, Xiamen, 361003 Fujian China; 2 Laboratory of Xiamen Cancer Center, The First Affiliated Hospital of Xiamen University, School of Medicine, Xiamen University, 55 Zhenhai Road, Xiamen, 361003 Fujian China; 3 State Key Laboratory of Cellular Stress Biology, School of Life Sciences, Xiamen University, Xiamen, 361100 Fujian China; 4 State Key Laboratory of Cellular Stress Biology, School of Life Sciences, Xiamen University, Xiamen, 361100 Fujian China

**Keywords:** NSCLC, OVOL2, NF-κB signaling, Glucose import

## Abstract

**Background:**

Tumor cells tend to utilize glycolysis rather than aerobic respiration even under aerobic conditions. OVOL2, an inhibitory C2H2 zinc finger transcription factor, is a potential tumor suppressor in cancers. However, the association between OVOL2 and tumor energy metabolism is unknown.

**Methods:**

Western blotting was used to determine the expression of OVOL2 in different non-small cell lung cancer (NSCLC) cell lines and mouse models. The metabolic parameters in NSCLC cells following overexpression or knockdown OVOL2 were examined. To define the mechanism by which OVOL2 regulates aerobic glycolysis, interacting protein of OVOl2 and downstream molecular events were identified by luciferase assay and co-immunoprecipitation. We documented the regulatory mechanism in mouse xenograft models. Finally, clinical relevance of OVOL2, NF-κB signaling and GLUT1 was measured by immunostaining.

**Results:**

OVOL2 is downregulated in NSCLC and overexpression of OVOL2 inhibits the survival of cancer cells. Moreover, OVOL2 directly binds to P65 and inhibits the recruitment of P300 but facilitates the binding of HDAC1 to P65, which in turn negatively regulates NF-κB signaling to suppress GLUT1 translocation and glucose import. In contrast, OVOL2 expression is negatively regulated by NF-κB signaling in NSCLC cells via the ubiquitin–proteasome pathway. Re-expression of OVOL2 significantly compromise NF-κB signaling-induced GLUT1 translocation, aerobic glycolysis in NSCLC cells and mouse models. Immunostaining revealed inverse correlations between the OVOL2 and phosphorylated P65 levels and between the OVOL2 and membrane GLUT1 levels, and a strong correlation between the phosphorylated P65 and membrane GLUT1 levels.

**Conclusions:**

These results suggest a regulatory circuit linking NF-κB and OVOL2, which highlights the role of NF-κB signaling and OVOL2 in the modulation of glucose metabolism in NSCLC.

**Video Abstract**

**Supplementary Information:**

The online version contains supplementary material available at 10.1186/s12964-022-00845-z.

## Background

Glucose catabolism involves anaerobic glycolysis and aerobic oxidation, among which glycolysis is the most important glucose metabolic pathway. One molecule of glucose is decomposed into two molecules of pyruvate by glycolysis, and two molecules of ATP are produced during this process. The fate of pyruvate varies with oxygen concentration. Under hypoxic conditions, pyruvic acid is reduced to lactic acid and transported out of cells, a process called anaerobic metabolism. In contrast, under aerobic conditions, pyruvate enters mitochondria and is decomposed into CO_2_ and water through the tricarboxylic acid cycle and oxidative phosphorylation, producing a large amount of ATP; this process is called aerobic metabolism. In many tumors, including lung cancer, cells tend to utilize glycolysis rather than aerobic respiration even under aerobic conditions to adapt to their environment and meet the demands of energy metabolism and synthesis reactions. In contrast to the active process in normal tissue cells, this switch from aerobic respiration to aerobic glycolysis, namely, the Warburg effect, is a hallmark of tumor cells [1, 2]. Notably, the premise of glycolysis is that glucose can be transported into cells in a timely manner, and glucose intake is completed by glucose transporters (GLUTs). The function and transport efficiency of GLUTs directly determine whether cells have enough glucose for glycolysis. Therefore, the total transport capacity of GLUTs is the main limiting factor of glycolysis [3]. Recently, the Warburg effect has been proven to play a key role in tumorigenesis and has become an important target for tumor therapy [4]. Many oncogenes and tumor suppressor genes are involved in this process. Akt, c-myc, RAS and HIF-1α can enhance the Warburg effect, while PTEN and p53 can inhibit it [4–7]. Although much important progress has recently been made in this field, the molecular mechanism underlying the Warburg effect is incompletely understood in many cancer cells, including those of lung cancer.

The NF-κB transcription factor family includes five members: RelA (P65), RelB, c-Rel, NF-κB1 (P50) and NF-κB2 (P52). These proteins dimerize to form the functional NF-κB complex. The homodimers and/or heterodimers formed by the two subunits of the NF-κB complex bind to a specific sequence (κB site) in the target gene to regulate its transcription. The most common dimer of NF-κB is the heterodimer composed of P65 and P50. In resting cells, IκB, the inhibitory unit of NF-κB, binds to NF-κB through its C-terminal specific ankyrin repeat motif and conceals the nuclear localization sequence (NLS) to prevent nuclear translocation of NF-κB. When cells are stimulated by extracellular signals, the IκB kinase (IKK) complex is activated. Consequently, IκB is phosphorylated and degraded via the ubiquitin–proteasome pathway, resulting in the release of NF-κB and exposure of the nuclear localization sequence. Free NF-κB is further activated by posttranslational modifications (phosphorylation, acetylation, and glycosylation) and rapidly translocates to the nucleus, where it recognizes specific DNA sequences, thus inducing the transcription of related genes [8]. NF-κB can regulate the transition between gene activation or inhibition by forming different complexes with CBP/P300 or HDAC1 [9, 10]. However, the regulation of this transition is unclear. The NF-κB signaling pathway is often activated in various types of human cancer and plays a key role in tumorigenesis. Signal transduction via activated NF-κB has been reported to be able to promote the Warburg effect in cancer cells, but the underlying mechanism is unclear [11–13]. A recent report indicated that activation of NF-κB promotes the transport of GLUT1 to the plasma membrane; this may be an important mechanism by which NF-κB promotes the Warburg effect in cancer cells [14].

The *Ovo* gene encodes a set of evolutionarily conserved C2H2 zinc finger transcription factors. *Ovo* was first identified in Drosophila, in which it plays a very important role in epidermal development and egg formation [15, 16]. Its mouse and human homologs include Ovol1, Ovol2 and Ovol3. As Ovol3 is expressed only in early embryos, no studies focusing on this protein have been conducted. Current research focuses mainly on ovol1 and ovol2, whose main function is to inhibit cell proliferation and promote cell differentiation. Moreover, Ovol2 can maintain an epithelial differentiation status [17]. Many studies have shown that Ovol2 is closely related to epithelial–mesenchymal transition (EMT) during tumor invasion. Studies in breast cancer have found that Ovol2 inhibits EMT by direct transcriptional inhibition of ZEB1 expression [18], and studies in liver cancer have shown that Ovol2 inhibits EMT by indirectly promoting miR-200 expression [19], and that targeting TRPV1 can inhibit EMT by affecting the Ovol2-zeb1 axis [20]. A study in lung cancer found that Ovol2 inhibits EMT through direct transcriptional inhibition of Twist1 expression [21]. Moreover, one group found that in colorectal cancer, Ovol2 directly binds with Tcf4 and β-Catenin through protein–protein interactions to inhibit Wnt signaling and EMT [22], and our recent study showed that poly(ADP-ribosyl)ated Ovol2 plays an important role in regulating the chromosomal aneuploidy and death of cancer cells [23]. The above studies focused mainly on tissue development, and knowledge about the relationship of Ovol2 with tumors is mainly limited to its role in EMT. Research on other aspects of tumors, especially tumor energy metabolism, is lacking.

Lung cancer is one of the most severe malignancies and seriously threatens human health and quality of life. Non-small cell lung cancer (NSCLC), including adenocarcinoma, squamous cell carcinoma and large cell carcinoma, accounts for approximately 80% of all lung cancers and is the most common type of lung cancer. NSCLC typically has a poor prognosis and a high death rate; indeed, it is the leading cause of cancer-related death worldwide. Despite great advances in the diagnosis and treatment of NSCLC, the mortality rate remains high, especially in the advanced stage [24]. Therefore, there is a great need to identify new prognostic markers and develop novel therapeutic strategies for NSCLC. In the present study, we aimed to investigate the clinical relevance of NF-κB signaling and OVOL2 and examine their regulatory features in NSCLC cells and mouse tumor models. We identified a regulatory circuit connecting NF-κB and OVOL2, which highlights the role of NF-κB signaling and OVOL2 in the modulation of energy metabolism in NSCLC.

## Materials and methods

### Chemicals and antibodies

A Cell Counting Kit-8 (CCK-8, Cat# CK04) was purchased from Dojindo (Kumamoto, Japan). Pooled antisense oligonucleotides (i.e., siRNAs) against human OVOL2, P65, and GLUT1 were purchased from GenePharma (Shanghai, China). Antibodies against OVOL2 (Cat# ab101580) and P65 (Cat# ab16502) were purchased from Abcam (Cambridge, MA, USA). The following antibodies were used: Glycolysis Antibody Sampler Kits (Cell Signaling Technology; Cat# 12866 and Cat# 8337), anti-GLUT1, anti-GLUT2, anti-GLUT3, anti-GLUT4, anti-PGK1, anti-GCK, anti-PDP2, anti-DLD, anti-PCK1, anti-SDHA, anti-G6PD (all from Abcam; Cat# ab115730, ab234440, ab191071, ab188317, ab38007, ab184169, ab99170, ab133551, ab28455, ab14715, and ab210702, respectively), anti-GAPDH anti-Lamin B, anti-actin and anti-Calnexin (all from Cell Signaling Technology; Cat# 8884, 13435, 3700 and 2433, respectively).

### Cell culture

The human embryonic kidney cell lines HEK293T and HEK 293, the human bronchial epithelial cell line BEAS-2B, and the NSCLC cell lines NCI-H1299, HCC827, A549, NCI-H1650, SK-MES-1, H226, NCI-H661 and NCI-H460 were purchased from ATCC (Manassas, VA). Cells were cultured in Dulbecco’s modified Eagle’s medium (DMEM) supplemented with 10% fetal calf serum (Gibco), 100 units/ml penicillin, and 100 mg/ml streptomycin. All cell lines were grown at 37 °C in 5% carbon dioxide. The proteasome inhibitors MG132, CQ, and CHX were purchased from Sigma.

### Lentivirus-mediated RNA interference or gene transfer

The lentiviral pLKO.1 vector was used to express short hairpin RNA (shRNA) directed against either human OVOL2 or GLUT1 or a nonsilencing scrambled control sequence. The target sequences were as follows: OVOL2 shRNA1, 5′-GCTGGGATGAGCTCCCGGATGAGAA-3′; OVOL2 shRNA2, 5′-ACATCCGCACACCAGGAGAAT-3′; P65 shRNA1, 5′-CCTGAGGCTATAACTCGCCTA-3′; P65 shRNA2, 5′- CACCATCAACTATGATGAGTT-3′; GLUT1 shRNA1, 5′-CCAAAGTGATAAGACACCCGA-3′; GLUT1 shRNA2, 5′- CTTCAAAGTTCCTGAGACTAA-3′; and control shRNA, 5′- TTCTCCGAACGTGTCACGT-3′. For overexpression of OVOL2 and P65, the corresponding sequences were cloned into the lentiviral vector pLV-CS2.0 under the control of the EF1α promoter. Lentiviral vectors were produced by cotransfecting pLKO.1 or pLV-CS2.0 carrying the expression cassette with the helper plasmids pMD2.g and psPAX2 into HEK293T cells using Lipofectamine 3000 (Invitrogen Life Technology). The viral supernatant was collected 48 h after transfection, and the viral titer was determined by transduction of HeLa cells with serial dilutions of the supernatant and analysis of Colony Formation Titering Assay for Lentivirus. Cells at 50–70% confluence were transduced with the viral vectors in the presence of 10 mg/ml polybrene for 24 h. Fresh medium was then added to the transduced cells, which were later selected with puromycin.

### Cell proliferation assay

Cell proliferation was analyzed by a CCK-8 (Dojindo) assay following the manufacturer’s instructions. The absorbance was measured at 450 nm using a microplate reader.

### Real-time quantitative PCR (qPCR)

Total RNA (2 μg) from cells was used for first-strand cDNA synthesis (Invitrogen). Platinum SYBR Green qPCR SuperMix (Invitrogen) was used for qPCR, and the expression levels were quantified using the − ΔΔCt method. The primer sequences used to amplify OVOL2 were 5′-TCACCTCAAGTGCCACAACCA-3′ (forward) and 5′-TGTAGCCGCAATCCTCGCA-3′ (reverse), for ZEB1 were 5′-TCCATGCTTAAGAGCGCTAGCT-3′ (forward) and 5′-ACCGTAGTTGAGTAGGTGTATGCCA-3′ (reverse), for Skp2 were 5′-GGCAAAGGGAGTGACAAAGA-3′ (forward) and 5′-TCAAAGCACCAGGAGAGATT-3′ (reverse), and for Notch1 were 5′-ACCAGGAGCAGAGGACGTC-3′ (forward) and 5′-CTTTCCTGGCACACCTCTTG-3′ (reverse).

### Co-IP assay, glutathione S-transferase (GST) pulldown assay and Western blot analysis

Transfected cells were lysed with lysis buffer (20 mM Tris–HCl (pH 7.5), 150 mM NaCl, 1 mM EDTA (pH 8.0), 1 mM EGTA (pH 8.0), and 1% Triton X-100) for subsequent co-IP. The cell lysates were precleared with protein A/G beads for 1 h at 4 °C with agitation. Specific or control IgG antibodies were added to the precleared samples and incubated with rotation at 4 °C for 4 h or overnight. The immune complexes were captured with 20 μl of protein A/G beads at 4 °C for 1 h, washed three times with washing buffer and subjected to SDS-PAGE for subsequent Western blot analysis.

GST fusion proteins were expressed in the *E. coli* strain BL21. To purify the GST fusion proteins, cells were lysed by sonication in lysis buffer (phosphate-buffered saline [PBS], 1% Triton X-100, 2% β-mercaptoethanol, and 0.1 mM phenylmethylsulfonyl fluoride [PMSF]), and the resulting lysates were incubated for 1 h at 4 °C with glutathione-Sepharose beads. The beads were pelleted by centrifugation and washed with dialysis buffer for subsequent experiments. Nuclear extracts were then incubated with resin-bound proteins at 4 °C for 3 h with rotation, washed four times in washing buffer (20 mM HEPES (pH 7.9), 0.2 mM EDTA (pH 8.0), 20% glycerol, 0.15 M KCl, and 0.2% NP-40) and analyzed by Western blotting using the appropriate antibodies. Western blotting was performed according to standard procedures.

### HDAC and HAT activity assays

For the HDAC activity assay, cell lysates were incubated with an anti-P65 antibody and protein A/G beads for 3–5 h. The beads were washed 3 times with NP-40 lysis buffer and were then fixed with 85 μl of double-distilled H_2_O. Then, 10 μl of 10× HDAC assay buffer and 5 μl of HDAC colorimetric substrate were added to each well (Colorimetric HDAC Activity Assay Kit, Biovision, Milpitas, CA). The plates were incubated at 37 °C overnight. The reaction was stopped by adding 10 μl of lysine developer and mixed well at 37 °C for 1 h. The samples were analyzed at 405 nm in an enzyme-linked immunosorbent assay plate reader.

For the HAT activity assay, cell lysates were incubated with an anti-P65 antibody and protein A/G beads for 3–5 h. The beads were washed 3 times with NP-40 lysis buffer and were then fixed with 32 μl of double-distilled H_2_O. Then, 50 μl of 2× HAT assay buffer, 10 μl of HAT substrate and 8 μl of NADH generating enzyme were added to each well (Colorimetric HAT Activity Assay Kit, Biovision, Milpitas, CA). The plates were incubated at 37 °C for 4 h. The samples were analyzed at 440 nm in an enzyme-linked immunosorbent assay plate reader.

### NF-κB-specific luciferase assay

For the NF-κB-specific luciferase assays, BEAS-2B and NCI-H1650 cells were seeded in 6-well plates. The luciferase reporter plasmid and the indicated expression plasmids were cotransfected with Lipofectamine 3000. The transfection of equivalent total amounts of plasmid DNA was ensured by the addition of empty vector. Luciferase activities were measured 48 h after transfection using a Dual-Luciferase Assay kit (Promega) on a TriStar^2^ LB 942 Multimode Reader (Berthold Technologies, Bad Wildbad, Germany) following the manufacturer’s instructions. pRL-TK was cotransfected as the internal control. Experiments were performed in triplicate.

### Measurement of glucose uptake, lactate release, ATP levels, and O_2_ consumption

Intracellular glucose was measured using cell lysates and a glucose assay kit (BioVision), extracellular lactate was measured using cell culture medium and a lactate assay kit (BioVision), ATP levels were measured using an ATP assay kit (Promega), and oxygen consumption was evaluated using an oxygen consumption rate assay kit (Cayman Chemical) according to the manufacturers’ instructions. The measured values were normalized to the protein concentration.

### Immunohistochemical analysis

All fresh tissues were fixed with 10% neutral buffered formalin, stored in 70% ethanol, paraffin embedded and sectioned. After dewaxing and rehydration, antigen retrieval was performed by boiling in citrate buffer (pH 6.0) for 25 min. The sections were then pretreated with peroxidase blocking buffer for 20 min at room temperature. After treatment with blocking buffer (5% normal goat serum in PBS) for 1 h at room temperature, the sections were incubated with the primary antibody in blocking buffer. Secondary antibody reagents from a DAB kit were used.

### Aniamals

Four-week-old female BALB/c nude mice were used to conduct xenograft experiments. A total of 5 × 10^6^ cells were injected subcutaneously into the dorsal thighs of mice. Tumor growth was examined regularly for 6 weeks, and the tumor volume was calculated by each week. All mice were raised under specific pathogen-free conditions in Xiamen University Laboratory Animal Center (Xiamen University, China) in accordance with institutional guidelines. The research protocol was approved by the local Ethical Committee of Xiamen University.

### Patient tissue samples

Surgical resection samples from primary human NSCLC tissues and the corresponding adjacent normal tissues were used to detect the protein expression patterns of OVOL2, pP65, and GLUT1. All samples were obtained from the First Affiliated Hospital of Xiamen University with patient consent and institutional review board approval. These samples were subsequently de-identified to protect patient confidentiality.

### Statistical analysis

Data were analyzed using GraphPad Prism software. For tissue microarray analyses, Fisher’s exact test was used to compare qualitative variables; quantitative variables were analyzed using t tests. Data are presented as the means ± SDs. All data were analyzed using two-sided Student’s t tests, and *p* < 0.05 was considered statistically significant.

## Results

### OVOL2 downregulation in NSCLC promotes the survival of cancer cells

**Fig. 1:**
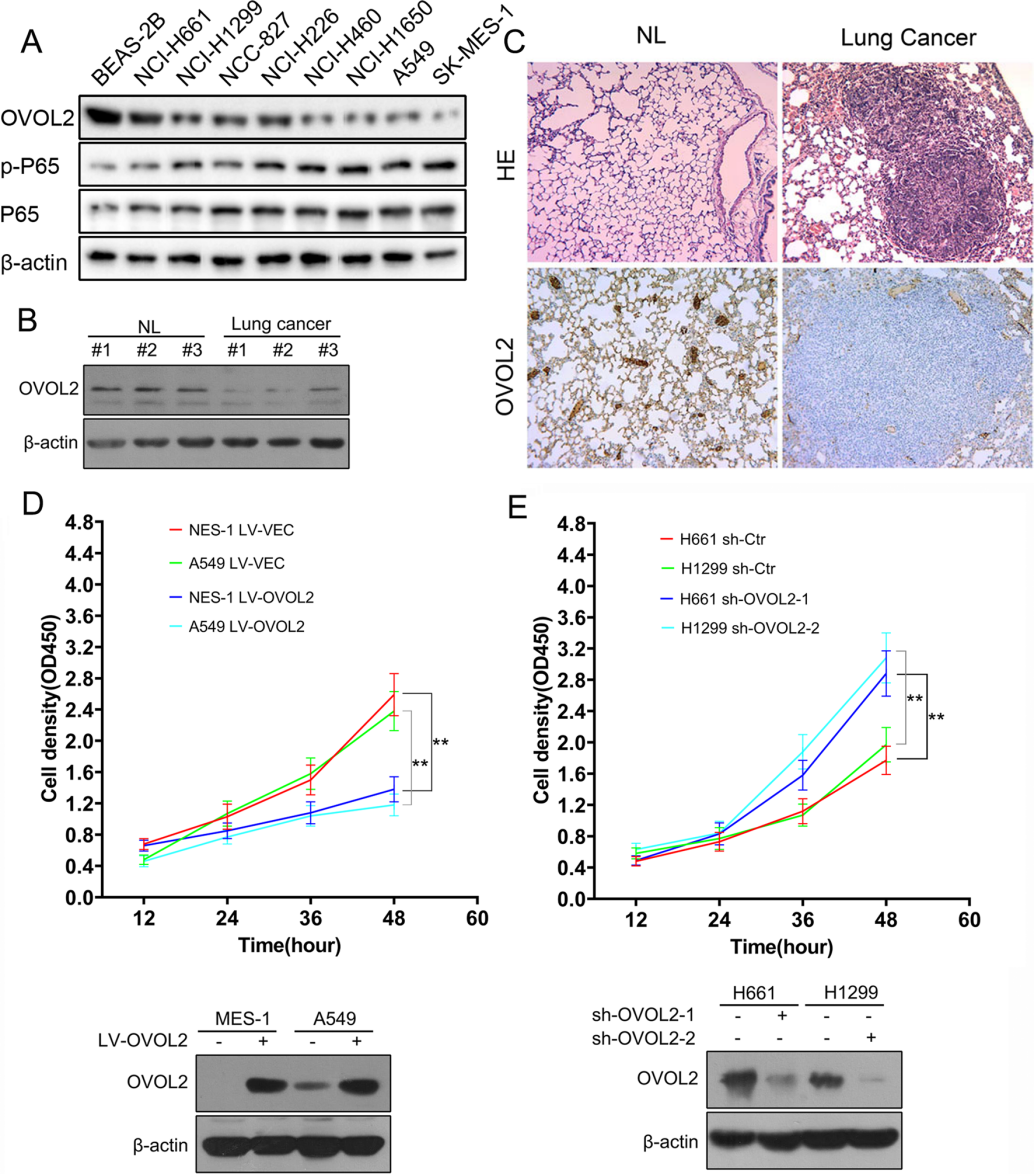
Expression pattern of OVOL2 and its effects on cell proliferation in NSCLC cells. **A** Western blot analysis of OVOL2 and pP65 in a variety of cell lines, including normal lung and NSCLC cells. **B** Western blot analysis of OVOL2 in lung cancer tissue derived from *Kras*^G12D^ mice versus normal lung tissue. **C** Immunohistochemical staining of OVOL2 in specimens obtained from *Kras*^G12D^ mice versus normal lung tissue. **D** Top, overexpression of OVOL2 suppressed the proliferation (n = 3) of SK-MES-1 and A549 cells. Bottom, Western blot analysis of OVOL2 in OVOL2-overexpressing SK-MES-1 and A549 cells. **E** Top, knockdown of OVOL2 enhanced the proliferation (n = 3) of NCI-H661 and NCI-H1299 cells. Bottom, Western blot analysis of OVOL2 in OVOL2-knockdown NCI-H661 and NCI-H1299 cells. The data were expressed as the means ± SDs. **, *p* < 0.01

To characterize the effect of OVOL2 on tumorigenesis in NSCLC, we determined the protein expression levels of OVOL2 in different NSCLC cell lines. Compared with the normal human bronchial epithelial cell line BEAS-2B, cell lines of the three types of NSCLC—lung adenocarcinoma (NCI-H1299, HCC827, NCI-H1650 and A549), squamous cell lung carcinoma (NCI-H226 and SK-MES-1) and large cell lung carcinoma (NCI-H661 and NCI-H460)—exhibited decreased expression levels of OVOL2. (Fig. 1A). Moreover, an inverse relationship between the protein levels of OVOL2 and phosphorylated P65 was observed in these NSCLC cell lines (Fig. 1A). We also examined the protein expression level of OVOL2 in a *Kras*^G12D^-based lung cancer mouse model. Through Western blot analysis, we found that OVOL2 expression was lower in murine lung cancer tissue derived from *Kras*^G12D^ mice than in normal lung tissue (Fig. 1B). The above result was also verified by immunohistochemistry (IHC) (Fig. 1C). Therefore, we hypothesized that OVOL2 may play an important role in NSCLC tumorigenesis. To further determine the physiological relevance of OVOL2 in NSCLC cells, we overexpressed OVOL2 in OVOL2 low expression SK-MES-1 and A549 cells and found that overexpression of OVOL2 significantly impaired the survival of these cells (Fig. 1D). In contrast, knockdown of OVOL2 in OVOL2 high expression NCI-H661 and NCI-H1299 cells resulted in a significant increase in cell survival (Fig. 1E). Taken together, these data demonstrate that OVOL2 is downregulated in NSCLC and can impair the survival of cancer cells.

### OVOL2 inhibits aerobic glycolysis in NSCLC cells

**Fig. 2:**
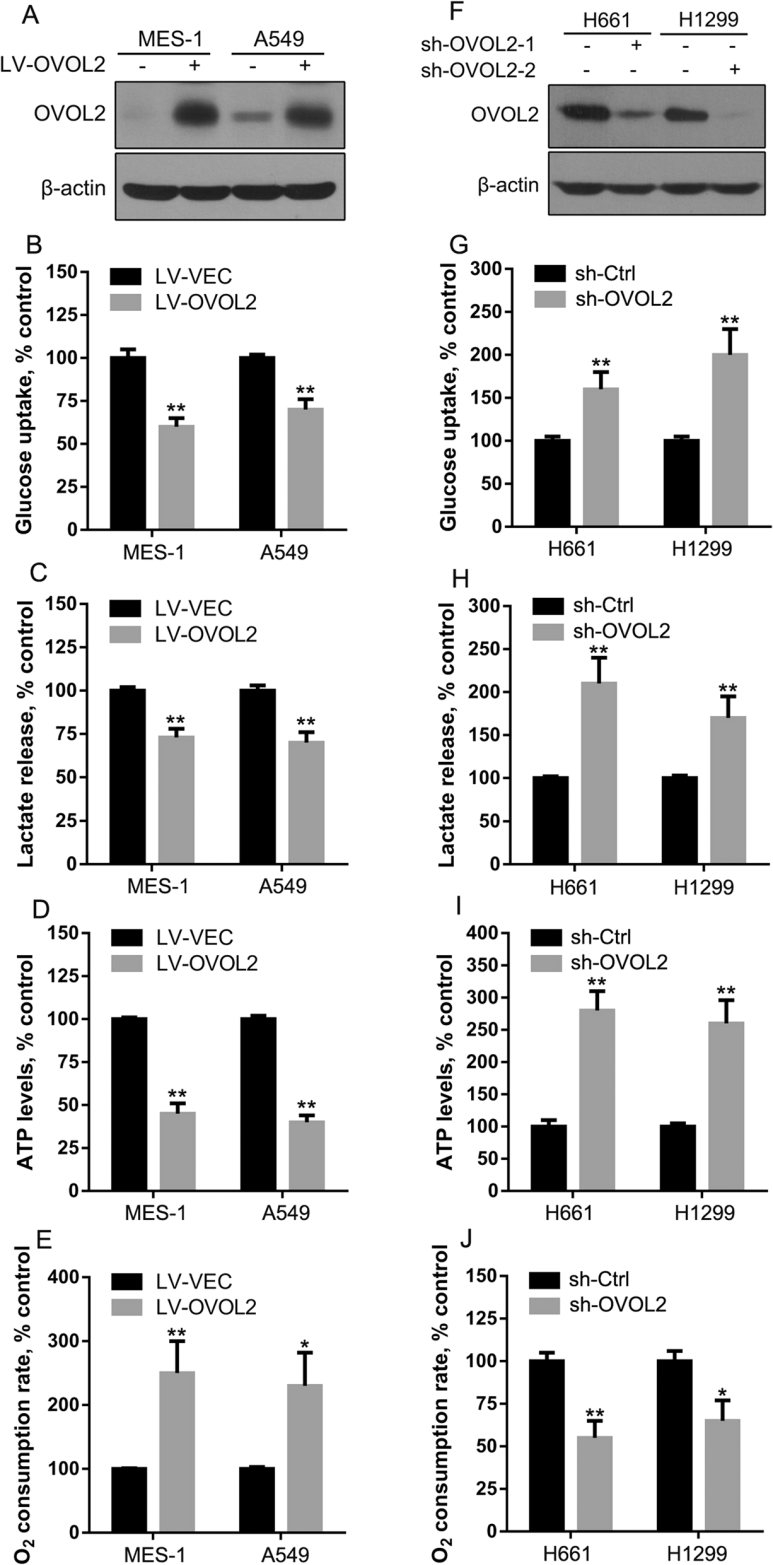
Effects of OVOL2 overexpression or knockdown on aerobic glycolysis in NSCLC cells. **A** Western blot analysis of OVOL2 in OVOL2-overexpressing SK-MES-1 and A549 cells. **B** Cellular glucose uptake, **C** lactate release, **D** the ATP level, and **E** the O_2_ consumption rate were measured in OVOL2-overexpressing SK-MES-1 and A549 cells. **F** Western blot analysis of OVOL2 in OVOL2-knockdown NCI-H661 and NCI-H1299 cells. **G** Cellular glucose uptake, **H** lactate release, **I** the ATP level, and **J** the O_2_ consumption rate were measured in OVOL2-knockdown NCI-H661 and NCI-H1299 cells. The data are expressed as the means ± SDs; n = 6 per group. *, *p* < 0.05; **, *p* < 0.01

Aberrant glucose metabolism has been identified in NSCLC cells. We hypothesized that OVOL2 may regulate aerobic glycolysis, a hallmark of cancer, to maintain NSCLC cell survival. To confirm this hypothesis, we overexpressed OVOL2 in OVOL2 low expression SK-MES-1 and A549 cells (Fig. 2A) and examined the metabolic parameters. Both cellular glucose uptake and lactate release into the culture medium were significantly decreased (Fig. 2B, C). In addition, OVOL2 overexpression led to reduced cellular ATP levels and enhanced cellular O_2_ consumption rates (Fig. 2D, E). In contrast, knockdown of OVOL2 in OVOL2 high expression NCI-H661 and NCI-H1299 cells (Fig. 2F) resulted in the opposite effects on the above metabolic parameters (Fig. 2G–I). Collectively, these results suggest that OVOL2 inhibits aerobic glycolysis in NSCLC cells.

### OVOL2 regulates aerobic glycolysis in NSCLC cells through GLUT1 mediated glucose import

**Fig. 3:**
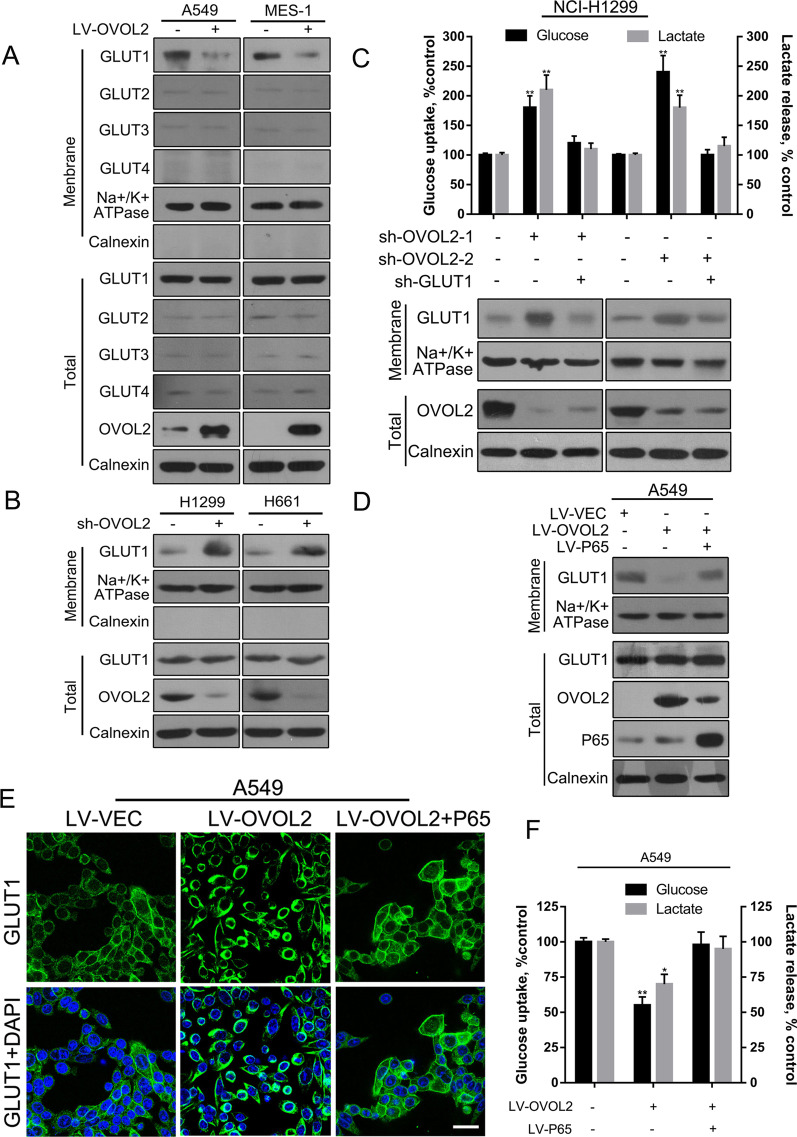
OVOL2 regulates aerobic glycolysis in NSCLC cells through the NF-κB/GLUT1 pathway. **A** Western blot analysis of GLUT proteins in OVOL2-overexpressing A549 and SK-MES-1 cells. **B** Western blot analysis of the GLUT1 protein in OVOL2-knockdown NCI-H1299 and NCI-H661 cells. **C** Cellular glucose uptake and lactate release levels were measured in OVOL2-knockdown NCI-H1299 cells after knockdown of membrane GLUT1 to the control level. **D** Western blot analysis of the GLUT1 protein in OVOL2-overexpressing A549 cells after overexpression of P65. **E** Assessment of GLUT1 localization by IF staining in the cells described in **D**. **F** Cellular glucose uptake and lactate release levels were measured in the cells described in **D**. For **C** and **F**, the data are expressed as the means ± SDs; n = 3 per group. *, *p* < 0.05; **, *p* < 0.01. Scale bar: 50 mm

To define the downstream molecular mechanism by which OVOL2 regulates aerobic glycolysis, we examined the protein expression of all critical enzymes and GLUTs involved in glucose metabolism in A549 and SK-MES-1 cells overexpressing OVOL2. As shown in Additional file 1: Fig. S1, none of the enzyme levels were significantly different between OVOL2-overexpressing and control cells. Therefore, we examined the expression status of the four GLUTs. Since GLUT proteins are transported from the cytoplasm to the plasma membrane to perform their functions, we evaluated both the plasma membrane and total expression levels of these proteins. OVOL2 did not affect the total protein level of any GLUT protein (Fig. 3A). However, overexpression of OVOL2 resulted in an obvious decrease in GLUT1 protein expression on the plasma membrane. However, the expression of the other three GLUT proteins was not significantly changed (Fig. 3A), suggesting that OVOL2 regulates GLUT1 via a membrane translocation-related mechanism. Consistent with this speculation, knockdown of OVOL2 in NCI-H1299 and NCI-H661 cells significantly increased the membrane GLUT1 protein level (Fig. 3B). Since GLUT1 mediates glucose import across the cell membrane, which is the first step of glycolytic metabolism in cancer cells, we next sought to determine whether GLUT1 is involved in OVOL2-regulated glycolysis inhibition. We knocked down OVOL2 expression in NCI-H1299 cells and observed the expected increase in membrane GLUT1 protein expression. However, when the membrane GLUT1 level was knocked down to the control level in the above cells, OVOL2 knockdown caused increases in glucose uptake, and lactate production were then brought down to control level (Fig. 3C). These findings suggest that OVOL2 regulates aerobic glycolysis in NSCLC cells through GLUT1 mediated glucose import.

### OVOL2 regulates GLUT1 membrane translocation and aerobic glycolysis in NSCLC cells through NF-κB signaling

A previous report demonstrated that NF-κB signaling activates aerobic glycolysis by facilitating the translocation of GLUT1 to the plasma membrane in lymphoma cells [14]. We hypothesized that OVOL2 may inhibit plasma membrane translocation of GLUT1 through its suppression of NF-κB signaling, which could be a critical mechanism by which OVOL2 inhibits aerobic glycolysis. First, we detected the effects of NF-κB signaling in NSCLC cells. Ectopic expression of P65 in pP65 low expression NCI-H661 cells greatly promoted the membrane translocation of endogenous GLUT1, as shown by Western blot analysis using the plasma membrane fraction isolated from cells (Additional file 1: Fig. S2A). In contrast, knockdown of P65 in pP65 high expression SK-MES-1 cells reduced the membrane translocation of endogenous GLUT1 in these cells (Additional file 1: Fig. S2B). These results suggest that NF-κB signaling regulates GLUT1 translocation in NSCLC cells. Then, OVOL2 was overexpressed in OVOL2 low expression A549 cells. As expected, ectopic expression of OVOL2 significantly inhibited the membrane translocation of endogenous GLUT1, as determined by Western blotting. However, simultaneous activation of NF-κB signaling by overexpression of P65 almost completely abolished the inhibitory effects of OVOL2 on GLUT1 membrane translocation (Fig. 3D). Notably, OVOL2 protein expression was decreased in P65-overexpressing cells, which suggests that OVOL2 protein expression may be downregulated by P65. In order to more intuitively display the distribution of GLUT1 in different compartments of cells, immunofluorescence (IF) staining assay in the above A549 cells was conducted. As shown in Fig. 3E, in untreated A549 control cells, GLUT1 showed a very clear staining in membrane and a weaker staining in cytoplasm. When OVOL2 was overexpressed, a very strong cytoplasm staining of GLUT1 was observed, whereas the membrane staining of GLUT1 could not be distinguished. However, additional overexpression of P65 in the above cells could counteract the effect of OVOL2 on GLUT1 localization. Finally, we investigated whether OVOL2 inhibits aerobic glycolysis through its suppression of NF-κB signaling in the above-mentioned cells. While ectopic expression of OVOL2 significantly inhibited aerobic glycolysis, simultaneous activation of NF-κB signaling by overexpression of P65 completely abolished the inhibitory effects of OVOL2 on glucose uptake and lactate production in cancer cells (Fig. 3F). These results strongly suggest that OVOL2 negatively regulates aerobic glycolysis through its inhibition of NF-κB signaling.

### OVOL2 negatively regulates the NF-κB signaling pathway through its interaction with P65

**Fig. 4:**
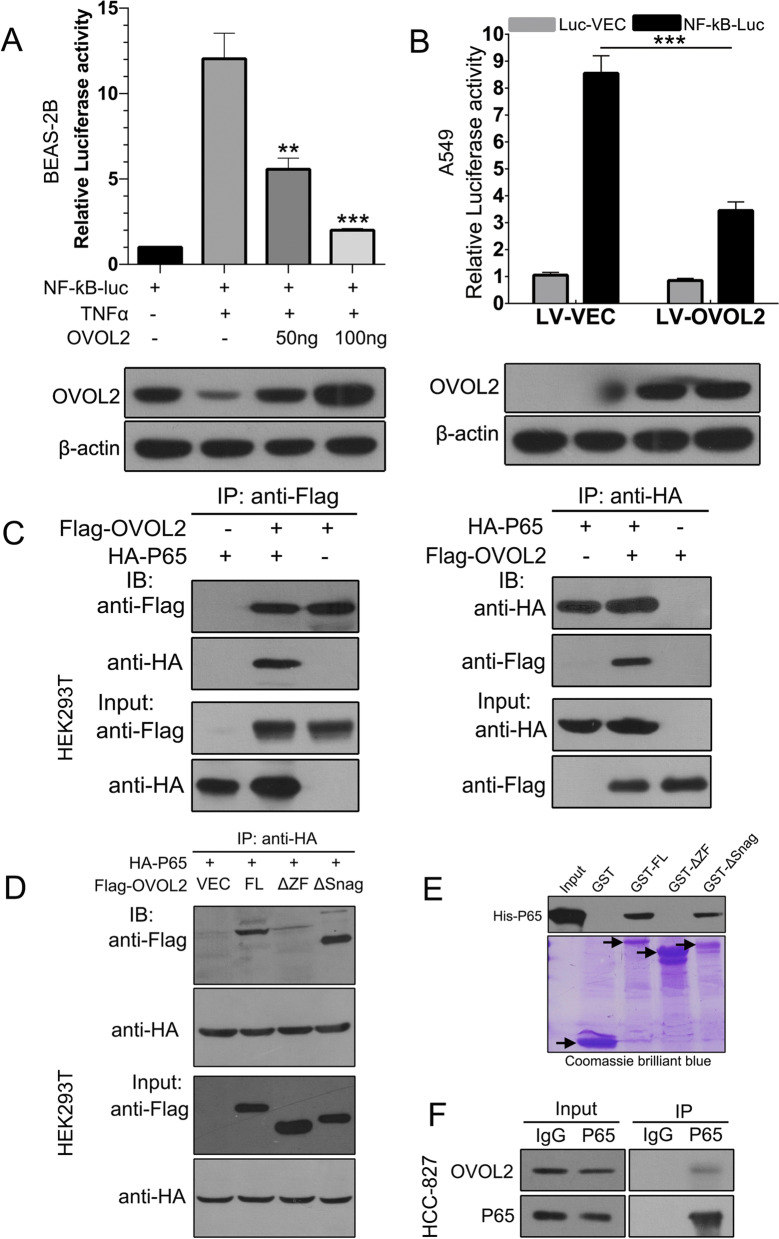
OVOL2 negatively regulates the NF-κB signaling pathway through its interaction with P65. **A** NF-κB-specific luciferase assay in OVOL2-overexpressing BEAS-2B cells stimulated with TNFα. BEAS-2B cells were transiently transfected with the indicated amount of plasmids and then NF-κB signaling was stimulated by adding recombinant TNFα protein to culture medium. Western blotting indicated OVOL2 expression. **B** NF-κB-specific luciferase assay in OVOL2-overexpressing A549 cells. Western blotting indicated OVOL2 expression. **C** Co-IP assay of HEK293T cells transfected with FLAG-tagged OVOL2 and HA-tagged P65. **D** Co-IP assay of HEK293T cells transfected with HA-tagged P65 and FLAG-tagged full-length, zinc finger-deleted or SNAG motif-deleted OVOL2. **E** In vitro pulldown assays of different recombinant proteins between His-tagged P65 and GST-tagged full-length, zinc finger-deleted or SNAG motif-deleted OVOL2. **F** Endogenous co-IP assay between OVOL2 and P65 in NCC-827 cells. For **A** and **B**, the data are expressed as the means ± SDs; n = 3 per group. **, *p* < 0.01 and ***, *p* < 0.001

Based on the above results, we sought to determine the mechanism by which OVOL2 regulates NF-κB signaling. To this end, we performed an NF-κB-specific luciferase assay in BEAS-2B cells. As shown in Fig. 4A, OVOL2 overexpression dose-dependently repressed transcriptional activation of the NF-κB-dependent luciferase reporter by TNFα. Notably, the luciferase assay in A549 cells transfected with NF-κB-luc indicated that the NF-κB-luc reporter was significantly activated, suggesting that endogenous NF-κB signaling is active in A549 cells. However, when OVOL2 was overexpressed, reporter activity was dramatically suppressed (Fig. 4B), supporting the role of OVOL2 in the inhibition of NF-κB signaling in NSCLC cells. To map the position in the NF-κB signaling pathway at which OVOL2 acts, we performed the following experiment. OVOL2 was overexpressed in SK-MES-1 cells or knocked down in NCI-H661 cells, and nuclear P65 protein expression was evaluated. As shown in Additional file 1: Fig. S3, the nuclear P65 protein level did not change upon either overexpression or knockdown of OVOL2, indicating that OVOL2 does not affect the nuclear translocation of P65. The above data suggest that OVOL2 does not act upstream of P65 in the cytoplasm, because any effect on those components would inhibit P65 nuclear translocation. Therefore, we speculated that OVOL2 regulates NF-κB signaling via a protein–protein interaction mechanism. To verify this hypothesis, we detected the association between OVOL2 and P65 via a coimmunoprecipitation (co-IP) assay in HEK293T cells transfected with FLAG-tagged OVOL2 and HA-tagged P65. As shown in Fig. 4C, OVOL2 indeed interacted with P65. The OVOL2 protein contains an N-terminal SNAG motif and a central region that contains four C2H2 zinc fingers. The co-IP assay also revealed that OVOL2 associates with P65 through its zinc finger domain (Fig. 4D). Next, an in vitro pulldown assay was performed, and the results indicated that OVOL2 directly associates with P65 (Fig. 4E). Finally, to investigate whether the endogenous OVOL2 and P65 proteins interact, we performed immunoprecipitation experiments using nuclear extracts from NCC-827 cells with moderation expression of both OVOL2 and pP65. As shown in Fig. 4F, immunoprecipitation with an anti-OVOL2 antibody also pulled down P65, suggesting that OVOL2 is typically present in a complex with P65 in cancer cells.

### OVOL2 inhibits the recruitment of P300 but facilitates the binding of HDAC1 to P65

**Fig. 5:**
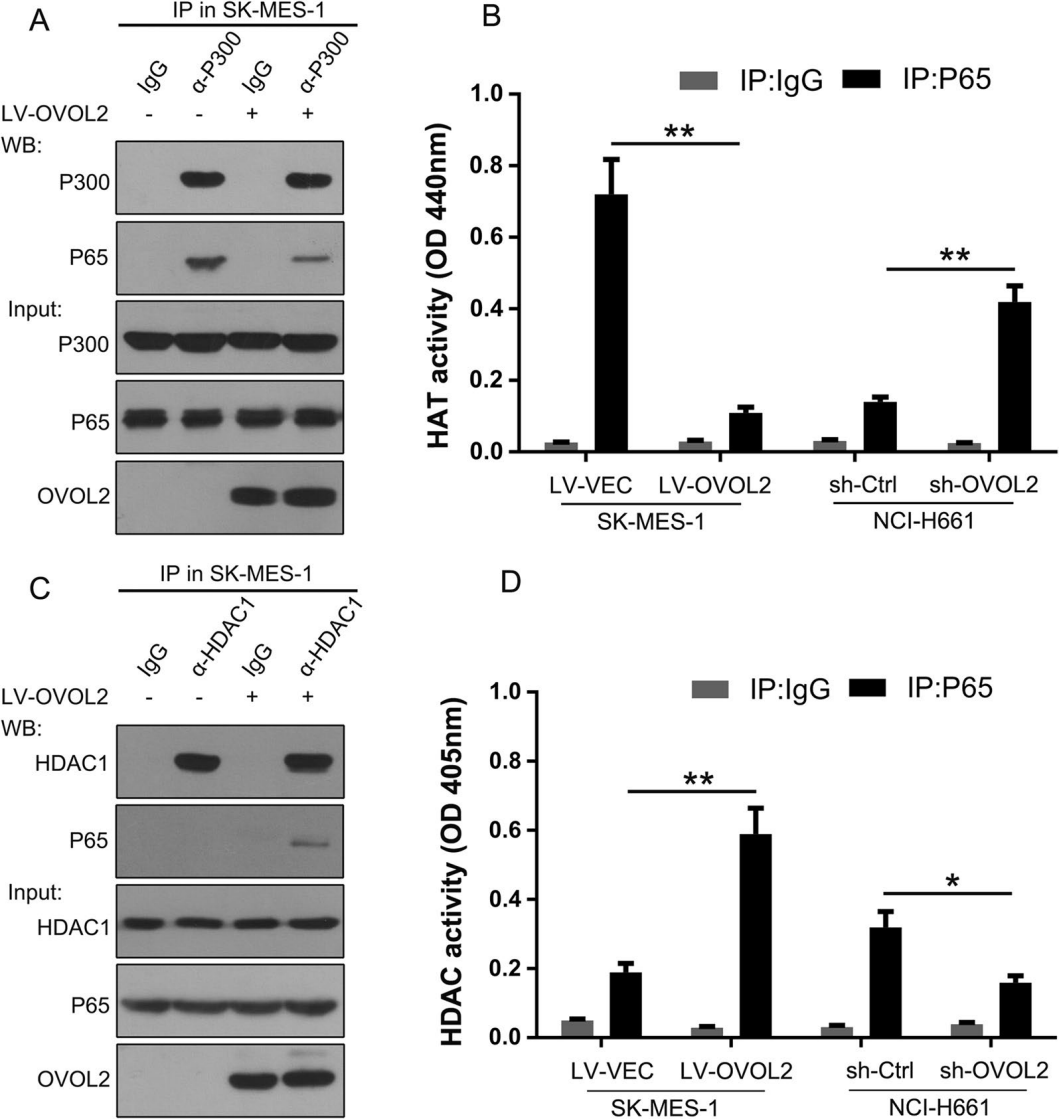
OVOL2 inhibits the recruitment of P300 but facilitates the binding of HDAC1 to P65. **A** Endogenous co-IP assay between P300 and P65 in SK-MES-1 cells with or without OVOL2 overexpression. **B** HAT activity assay using the P65 immunoprecipitate from SK-MES-1 cells with or without OVOL2 overexpression and from NCI-H661 cells with or without OVOL2 knockdown. The data are expressed as the means ± SDs of three independent experiments. **, *p* < 0.01. **C** Endogenous co-IP assay between P300 and HDAC1 in SK-MES-1 cells with or without OVOL2 overexpression. **D** HDAC1 activity assay using the P65 immunoprecipitate from SK-MES-1 cells with or without OVOL2 overexpression and from NCI-H661 cells with or without OVOL2 knockdown. The data are presented as the means ± SDs of three independent experiments. *, *p* < 0.05 and **, *p* < 0.01

A previous study indicated that recruitment of P300 is required for P65 to exert its transcriptional activity [9]. We thus sought to determine whether the association between P65 and P300 is affected by OVOL2. To test this hypothesis, a co-IP assay was performed in SK-MES-1 cells. As shown in Fig. 5A, P65 effectively recruited P300; however, in the presence of OVOL2, this recruitment was greatly inhibited. In the HAT activity assay with the P65 immunoprecipitate, overexpression of OVOL2 in SK-MES-1 cells resulted in significantly reduced HAT activity compared with that in the control cells, while knockdown of OVOL2 expression in NCI-H661 cells resulted in significantly enhanced HAT activity (Fig. 5B). A previous study demonstrated that OVOL2 recruits HDAC1 to repress gene expression [22]. We thus hypothesized that OVOL2 may facilitate the binding of HDAC1 to P65. As shown in Fig. 5C, P65 could not be recruited by HDAC1 in SK-MES-1 cells; however, when OVOL2 was overexpressed, the amount of HDAC1 coprecipitated with P65 was effectively increased. In the HDAC activity assay with the P65 immunoprecipitate, overexpression of OVOL2 in SK-MES-1 cells resulted in significantly enhanced HDAC activity compared with that in control cells, while knockdown of OVOL2 expression in NCI-H661 cells resulted in significantly reduced HDAC activity (Fig. 5D). Collectively, the above results suggest that OVOL2 regulates the formation of complexes between P65 and its coactivator/repressor, which in turn represses P65-mediated transcriptional activation and NF-κB signaling.

### OVOL2 protein expression is downregulated by P65 in NSCLC cells

**Fig. 6:**
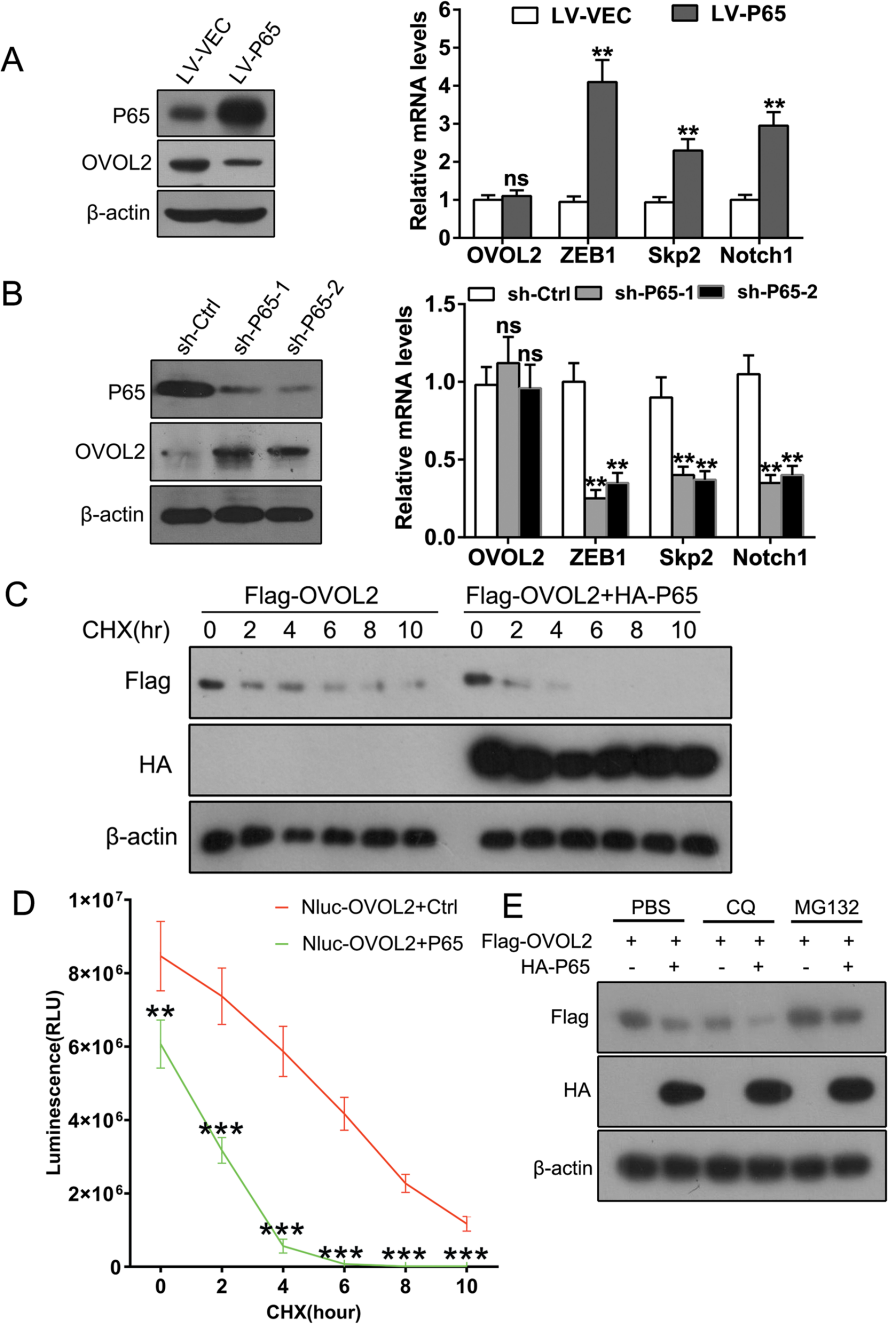
OVOL2 protein expression is downregulated by P65 in NSCLC cells. **A** NCI-H661 cells were transduced with empty lentiviral vectors or lentiviral P65 expression vectors. The protein levels of P65 and OVOL2 were evaluated by Western blot analysis (left), and the mRNA expression levels of OVOL2 and OVOL2 target genes (ZEB1, Skp2 and Notch1) were measured by qPCR (right). **B** SK-MES-1 cells stably expressing Ctrl shRNA or one of two different P65 shRNAs were generated. The protein levels of P65 and OVOL2 were evaluated by Western blot analysis (left), and the mRNA levels of OVOL2, ZEB1, Skp2 and Notch1 were measured by qPCR (right). **C** NCI-H661 cells were cotransfected with Flag-tagged OVOL2 and HA-tagged P65 or Ctrl vector, and a CHX chase assay was conducted. **D** NCI-H661 cells were cotransfected with Nluc-OVOL2 and pLV-P65 or Ctrl vector, and a NanoLuc luciferase assay was conducted to assess protein stability. **E** NCI-H661 cells were co-transfected with Flag-tagged OVOL2 and HA-tagged P65 or Ctrl vector. The cells were then treated with PBS (Ctrl), the proteasome inhibitor MG132 or the autophagy inhibitor CQ. OVOL2 protein stability was evaluated by Western blot analysis. In **A**, **B**, and **D**, the data are expressed as the means ± SDs of three independent experiments. **, *p* < 0.01; ***, *p* < 0.001

Since an inverse relationship between the protein levels of OVOL2 and phosphorylated P65 was observed in NSCLC cell lines (Fig. 1A) and OVOL2 protein expression was decreased in P65-overexpressing cells (Fig. 3D), we speculated that P65 may regulate OVOL2 expression. We overexpressed P65 in NCI-H661 cells or knocked down P65 in SK-MES-1 cells and found that the protein level but not the mRNA level of OVOL2 was negatively regulated by P65 (Fig. 6A, B). Consequently, the OVOL2 downstream target genes ZEB1, Skp2, and Notch1 were also positively regulated by P65 (Fig. 6A, B). To further determine whether P65 affects the stability of OVOL2, we cotransfected HA-P65 and Flag-OVOL2 into HEK293T cells and treated the cells with the protein synthesis inhibitor cycloheximide (CHX). As shown in Fig. 6C, the half-life of OVOL2 was significantly reduced from approximately 8 h to approximately 3 h upon P65 overexpression. Similar results were observed in the Nano-Luc luciferase assay performed to assess protein stability (Fig. 6D). Therefore, P65 reduces the stability of OVOL2 by enhancing its protein degradation. Protein degradation is typically mediated by the ubiquitin–proteasome and autophagy pathways. We treated cells with the proteasome inhibitor MG132 and the autophagy inhibitor chloroquine (CQ) and found that MG132 significantly inhibited OVOL2 protein degradation mediated by P65 (Fig. 6E). Thus, we concluded that OVOL2 protein expression is downregulated by P65 in NSCLC cells via degradation through the ubiquitin–proteasome pathway. The above results imply that removal of OVOL2-mediated suppression might be essential for the function of NF-κB signaling in NSCLC cells.

### The expression patterns of OVOL2 and GLUT1 are critical for NF-κB signaling-induced NSCLC cell survival

**Fig. 7:**
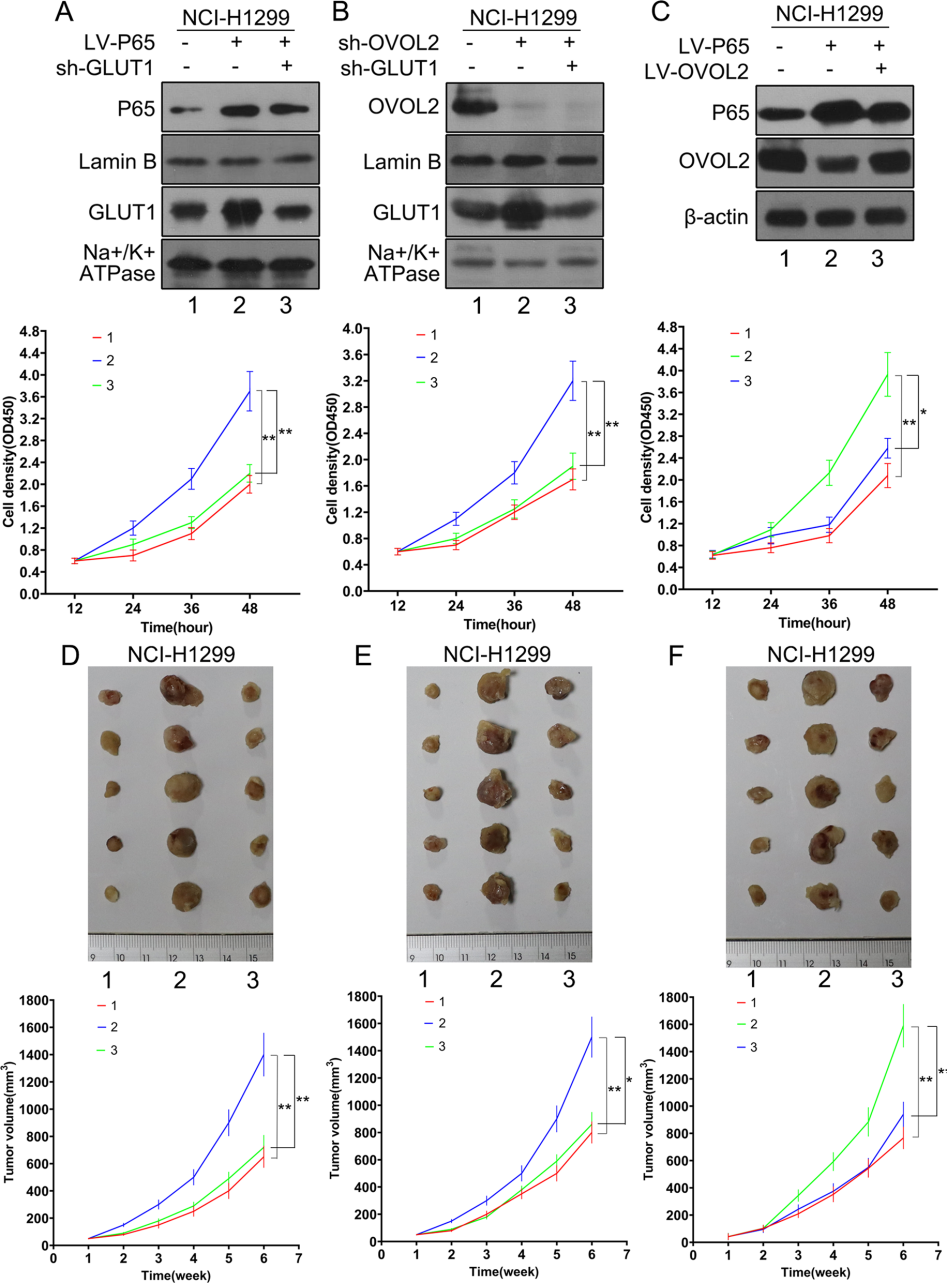
The expression patterns of OVOL2 and GLUT1 are critical for NF-κB signaling-induced NSCLC cell survival. **A** Cell proliferation was assessed in P65-overexpressing NCI-H1299 cells after knockdown of membrane GLUT1 to the control level. **B** Cell proliferation was assessed in OVOL2-knockdown NCI-H1299 cells after knockdown of membrane GLUT1 to the control level. **C** Cell proliferation was assessed in P65-overexpressing NCI-H1299 cells after reexpression of OVOL2 to the control level. **D**, **E**, **F** Photographs and growth curves of NCI-H1299 xenografts in nude mice. A total of 4 × 10^6^ cells described in **A**, **B**, and **C** were injected subcutaneously into mice. Tumor growth was monitored regularly for 7 weeks, and the tumor volume was calculated weekly. The data are expressed as the means ± SDs; n = 6 per group. *, *p* < 0.05; **, *p* < 0.01

Since our results clearly demonstrated that OVOL2 inhibits NF-κB signaling-induced GLUT1 membrane expression and aerobic glycolysis and that OVOL2 is downregulated by NF-κB signaling in NSCLC cells, we further explored whether the expression patterns of OVOL2 and GLUT1 are critical for NF-κB signaling-induced NSCLC cell survival. First, we knocked down GLUT1 in P65-overexpressing and OVOL2-knockdown NCI-H1299 cells. We chose NCI-H1299 cells because this cell line expresses lower pP65 and higher OVOL2. The cell growth assay revealed that knockdown of GLUT1 diminished the promoting effects of P65 overexpression and OVOL2 knockdown on cell survival (Fig. 7A, B), suggesting that GLUT1-mediated aerobic glycolysis is required for NF-κB signaling- and OVOL2-regulated NSCLC cell survival. Next, we overexpressed P65 in NCI-H1299 cells, and as expected, OVOL2 protein expression was downregulated. When we reexpressed OVOL2 in P65-overexpressing cells, the cell growth promotion mediated by P65 was inhibited (Fig. 7C). Next, xenograft experiments in nude mice were conducted by using the above cells. The size of tumors formed from P65-overexpressing or OVOL2-knockdown NCI-H1299 cells was significantly increased compared with the size of tumors formed from control cells. However, when GLUT1 was knocked down, tumor growth promoted by P65 overexpression or OVOL2 knockdown was obviously suppressed (Fig. 7D, E). Similarly, reexpression of OVOL2 significantly rescued the tumor growth phenotype driven by P65 overexpression in NCI-H1299 cells. These results demonstrate that removal of the suppressive effects of OVOL2 and membrane translocation of GLUT1 are critical for NF-κB signaling-induced NSCLC cell growth both in vitro and in vivo.

### Clinical relevance of OVOL2, NF-κB signaling and GLUT1

**Fig. 8:**
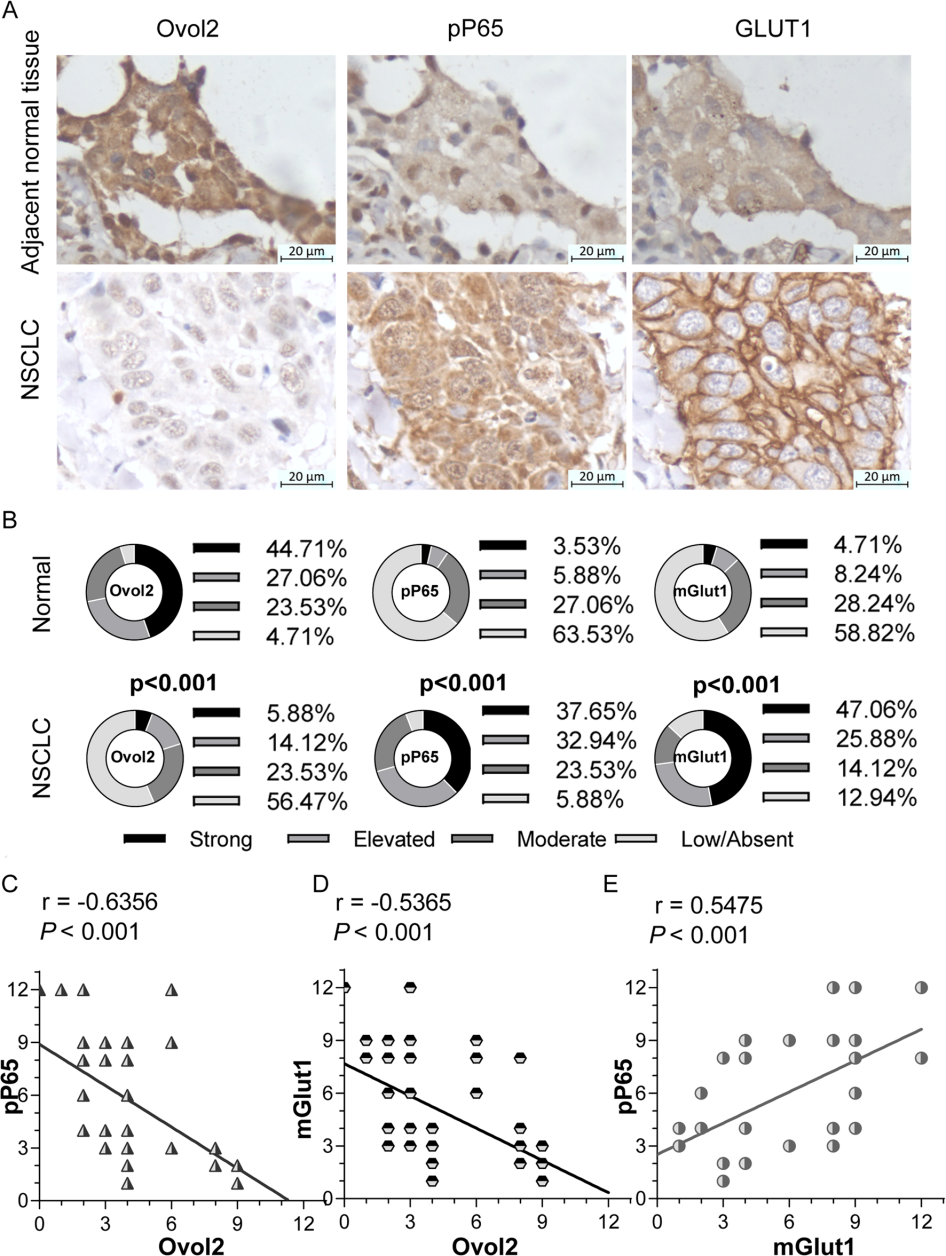
Clinical relevance of OVOL2, NF-κB signaling and GLUT1. **A** Representative immunostaining of OVOL2, pP65 and GLUT1 in NSCLC tissue samples and the corresponding adjacent normal tissue samples. Scale bars: 20 μm. **B** Relative percentages of the immunoreactivity scores for OVOL2, pP65 and membrane GLUT1 staining in 85 NSCLC tissue samples and the corresponding adjacent normal tissues. Fisher’s exact test was used to analyze categorical variables (*p* < 0.001). **C** Correlation between OVOL2 and pP65 protein levels. **D** Correlation between OVOL2 and membrane GLUT1 protein levels. **E** Correlation between pP65 and membrane GLUT1 protein levels. Pearson correlation analysis was performed on the data in **C** to **E** (r and *p* values are shown on the corresponding plots)

To investigate whether OVOL2 is associated with NF-κB signaling and GLUT1 expression in NSCLC patients, 85 NSCLC tissue samples and the corresponding adjacent normal tissues were analyzed by immunostaining to detect the expression patterns of OVOL2, phosphorylated P65, and GLUT1. As indicated by the representative samples shown in Fig. 8A, the overall protein expression levels of OVOL2 were significantly lower in NSCLC tissues than in adjacent normal tissues, whereas the phosphorylated P65 and membrane GLUT1 protein expression levels were significantly higher in NSCLC tissues. A quantitative summary of all samples is shown in Fig. 8B. Correlation analysis revealed inverse correlations between the OVOL2 and phosphorylated P65 levels (r = − 0.578, *p* < 0.001, Fig. 8C) and between the OVOL2 and membrane GLUT1 levels (r = − 0.642, *p* < 0.001, Fig. 8D) and a strong correlation between the phosphorylated P65 and membrane GLUT1 levels (r = 0.649, *p* < 0.001, Fig. 8E). Taken together, the above results show the clinical relevance of OVOL2, NF-κB signaling and GLUT1.

## Discussion

Previous studies have confirmed that OVOL2 plays an important role in tumor development and metastasis, including in lung cancer. However, the function of OVOL2 in regulating glucose metabolism in lung cancer cells has not been elucidated. Therefore, identifying the molecular mechanism by which OVOL2 regulates this process is crucial for understanding the biological functions of OVOL2 in lung cancer. In the present study, we showed that the expression of OVOL2 is downregulated in NSCLC. OVOL2 can inhibit aerobic glycolysis in NSCLC cells through suppression of GLUT1 membrane expression, a process mediated through the NF-κB signaling pathway. Furthermore, we found that OVOL2 inhibits NF-κB signaling by binding with P65 to regulate the transition between the NF-κB–P300 and NF-κB-HDAC1 complexes. As a transcription factor, OVOL2 usually regulates gene expression through directly binding to a gene promoter region. In a few cases, however, protein–protein interactions have been shown to be involved in the regulation of gene expression [22], a finding that is consistent with our observation and substantiates the multiple functions of OVOL2. Notably, we observed OVOL2 overexpression led to reduced cellular ATP levels but enhanced cellular O2 consumption rates (Fig. 2D, E). These two results seems to be paradoxical, because enhanced cellular O2 consumption would suggest an upregulation of oxidative phosphorylation, which should produce more ATP per molecule of glucose compared to glycolysis. We deduced that in our context, OVOL2 overexpression severely suppressed glycolysis and dramatically reduced ATP production via glycolysis. However, such reduction of ATP could be only partially compensated by relative increase of oxidative phosphorylation of fatty acids or amino acids catabolism. This hypothesis still needs to be further investigated.

The Warburg effect is one of the most important characteristics of cancer cells. The switch from oxidative phosphorylation to glycolysis is a key and unique mode of action by which cancer cells meet their rapidly increasing energy and biosynthesis requirements to support their rapid proliferation and survival. Further understanding of the mechanism underlying the Warburg effect is anticipated to provide new targets for cancer treatment. Recent studies have shown that many signaling pathways are involved in the metabolic switch of cancer cells to glycolysis. Changes in other genes during tumorigenesis also play a key role in regulating the Warburg effect in cancer cells [4, 25]. For example, the NF-κB signaling pathway has been shown to be frequently overactivated in many human cancers, including lung cancer, playing a very important role in the occurrence and development of cancers [8, 26, 27]. Previous studies have also demonstrated that activated NF-κB signaling can promote the Warburg effect in cancer cells, although the underlying mechanism is unclear [13]. A recent study in lymphoma indicated that NF-κB activation can promote the transport of GLUT1 to the plasma membrane, which may be an important mechanism by which NF-κB promotes the Warburg effect in cancer cells [14]. Many downstream targets of the NF-κB signaling pathway have been identified in different biological contexts; however, the exact downstream events and regulatory processes involved in glucose metabolism are unclear. In addition, although interactions among the components of this signaling pathway and some related proteins have been studied in detail, our knowledge of the mechanism by which the core component of NF-κB signaling is regulated remains obscure. In this paper, we studied the relationship between the NF-κB pathway and OVOL2. Our data showed that OVOL2 protein expression is negatively regulated by the NF-κB pathway through the ubiquitin–proteasome pathway. This result suggests that downregulation of OVOL2 is required for the function of NF-κB in glucose metabolism in NSCLC cells. The drawback of this part of the study is that E3 ubiquitin ligase, which mediates OVOL2 degradation, is still unknown. Future work will focus on the identification of the E3 ubiquitin ligase and the development of related inhibitors to improve the protein expression of OVOL2 in cancer cells and hence inhibit NF-κB pathway. Consequently, the growth of cancer cells can be effectively inhibited.

**Fig. 9:**
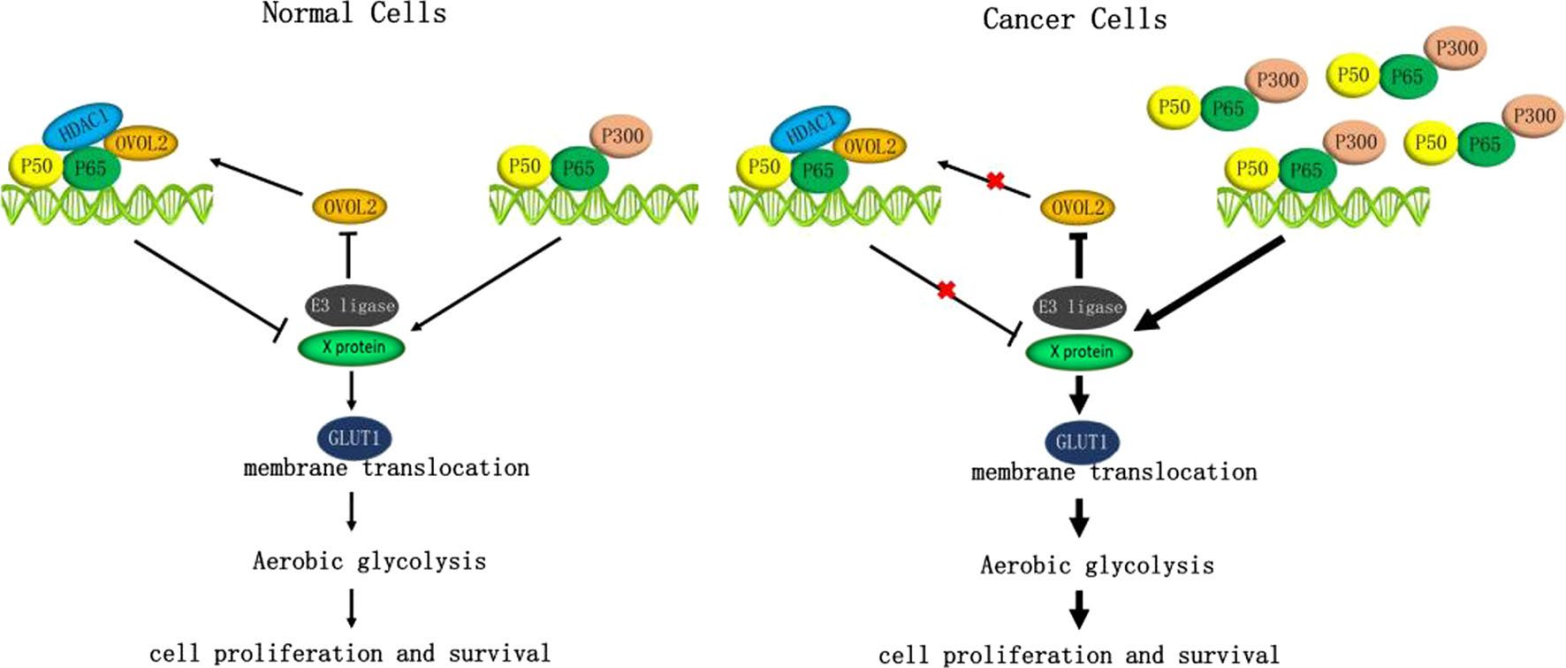
A schematic diagram summarizing the working model of the regulatory circuit between NF-κB and OVOL2. Left: in normal cells. Right: in Cancer cells. Two complexes—P50/P65–P300 and P50/P65–OVOL2–HDAC1—can potentially occupy the promoters of P65 target genes. Solid lines indicate the activated signaling events. Crossed lines indicate the inhibited signaling events

The reciprocal regulation between NF-κB and OVOL2 led us to conclude that NF-κB signaling and OVOL2 can mutually inhibit each other and form a regulatory circuit, i.e., a double negative feedback loop. On the one hand, NF-κB signaling negatively regulates OVOL2 through the ubiquitin–proteasome degradation pathway; on the other hand, OVOL2 inhibits NF-κB activity by binding with P65 to inhibit its recruitment of P300 and facilitate its recruitment of HDAC1, thus reducing the membrane transport of GLUT1 and finally inhibiting the Warburg effect. Considering all of the data collected in the investigation of this mechanism, we propose the mechanistic model shown in Fig. 9: Two complexes—P50/P65–P300 and P50/P65–OVOL2–HDAC1—can potentially occupy the promoters of P65 target genes. In normal lung epithelial cells, OVOL2 protein expression in the nucleus is relatively high to control the output of NF-κB signaling. Thus, glucose metabolism is maintained at a relatively low level in these cells, and their proliferation and survival can be precisely controlled. When cells become cancerous, however, NF-κB signaling is abnormally activated, and the nuclear P65 level is increased. Through a target E3 ubiquitin ligase protein, NF-κB can degrade the OVOL2 protein, resulting in relieve of inhibition by OVOL2. Consequently, the NF-κB signaling output level continues to increase, promoting glycolysis and maintaining the proliferation and survival of cancer cells.

Given the strong evidence for the regulation of GLUT1 protein expression by OVOL2 through NF-κB signaling, we sought to determine whether OVOL2, NF-κB signaling and GLUT1 are correlated in primary human NSCLC specimens. We analyzed 85 pairs of NSCLC samples along with the corresponding adjacent normal tissues by immunostaining to detect the expression of OVOL2, phosphorylated P65, and membrane GLUT1. Indeed, we observed a strong inverse correlation between the OVOL2 level and both the phosphorylated P65 and membrane GLUT1 levels and a strong correlation between the phosphorylated P65 and membrane GLUT1 levels, consistent with the results of our in vitro experiments.

## Conclusions

In conclusion, we identified a double negative feedback mechanism that can control the expression of NF-κB and OVOL2 both in vivo and in vitro, providing new insight into the regulation of NSCLC cell biological behaviors. Since NF-κB is a dominant oncogene in NSCLC and plays an important role in glucose metabolism homeostasis, manipulating the interaction between NF-κB and OVOL2 may constitute a new therapeutic approach for lung cancer.

## Supplementary Information

**Additional file 1.** **Figure S1.** The protein expression of all critical enzymes involved in glucose metabolism in A549 and SK-MES-1 cells overexpressing OVOL2. **Figure S2.** P65 promotes the membrane translocation of endogenous GLUT1. **Figure S3.** Nuclear P65 protein level is not regulated by OVOL2.

## Data Availability

All data generated or analyzed in this study are included in this published paper and the Additional information files. I can confirm I have included a statement regarding data and material availability in the declaration section of my manuscript.

## Abbreviations

CCK-8: Cell Counting Kit-8
CHX: Cycloheximide
Co-IP: Coimmunoprecipitation
CQ: Chloroquine
DAB: 3, 3′-Diaminobenzidine tetrahydrochloride
DLD: Dihydrolipoamide dehydrogenase
EDTA: Ethylenediaminetetraacetic acid
EGTA: Ethylene glycol tetraacetic acid
EMT: Epithelial–mesenchymal transition
G6PD: Glucose-6-phosphate dehydrogenase
GAPDH: Glyceraldehyde-3-phosphate dehydrogenase
GCK: Glucokinase
GLUT1: Glucose transporter type 1
GLUT1: Glucose transporter member 1
GLUT2: Glucose transporter member 2
GLUT3: Glucose transporter member 3
GLUT4: Glucose transporter member 4
GLUTs: Glucose transporters
GST: Glutathione S-transferase
HAT: Histone acetyltransferase
HDAC: Histone deacetylase
HDAC1: Histone deacetylase 1
HEPES: 4-(2-Hydroxyethyl)-1-piperazineethanesulfonic acid
HIF-1α: Hypoxia-inducible factor 1 alpha
IF: Immunofluorescence
IHC: Immunohistochemistry
IKK: IκB kinase
NADH: Nicotinamide Adenine Dinucleotide Hydride
NF-κB: Nuclear factor kappa B
NF-κB1: Nuclear factor kappa B subunit 1
NF-κB2: Nuclear factor kappa B subunit 2
NLS: Nuclear localization sequence
Notch1: Notch Receptor 1
NSCLC: Non-small cell lung cancer
OVOL2: Ovo Like Zinc Finger 2
p53: Tumor Protein P53
PBS: Phosphate-buffered saline
PCK1: Phosphoenolpyruvate carboxykinase 1
PDP2: Pyruvate dehyrogenase phosphatase catalytic subunit 2
PGK1: Phosphoglycerate kinase 1
PMSF: Phenylmethylsulfonyl fluoride
PTEN: Phosphatase and Tensin Homolog
qPCR: Quantitative real-time PCR
SDHA: Succinate dehydrogenase complex flavoprotein subunit A
Skp2: S-Phase Kinase Associated Protein 2
Tcf4: Transcription factor 4
TRPV1: Transient receptor potential cation channel subfamily V member 1
Twist1: Twist family bHLH transcription factor 1
ZEB1: Zinc finger E-box binding homeobox 1
