# Supplementary material for: An NF-κB/OVOL2 circuit regulates glucose import and cell survival in non-small cell lung cancer
Source: Cell Commun Signal. 2022 Mar 28;20:40. doi: 10.1186/s12964-022-00845-z (PMC8962559; doi:10.1186/s12964-022-00845-z)
Supplement: Supplementary file 2 — Additional file 1. Figure S1. The protein expression of all critical enzymes involved in glucose metabolism in A549 and SK-MES-1 cells overexpressing OVOL2. Figure S2. P65 promotes the membrane translocation of endogenous GLUT1. Figure S3. Nuclear P65 protein level is not regulated by OVOL2. [file 12964_2022_845_MOESM2_ESM.docx]

**An NF-κB/OVOL2 circuit regulates glucose import and cell survival in non-small cell lung cancer**

Rui Zhang^1, *^, Guo-Jun Geng^1, *^, Jian-Guang Guo^3, *^, Yan-Jun Mi^1^, Xiao-Lei Zhu^1^, Ning Li^1^, Hong-Ming Liu^1^, Jun-Feng Lin^1^, Jian-Weng Wang^1^, Guang Zhao^1^, Guan-Zhi Ye^1^, Bo-An Li ^3, #^, Qi-Cong Luo^2, #^, and Jie Jiang^1, #^

1. Department of Thoracic Surgery and Xiamen Cell Therapy Research Center, The First Affiliated Hospital of Xiamen University, School of Medicine, Xiamen University, Xiamen 361003, Fujian, China.

2. Laboratory of Xiamen Cancer Hospital, The First Affiliated Hospital of Xiamen University, School of Medicine, Xiamen University, Xiamen, 361003, Fujian, China.

1. State Key Laboratory of Cellular Stress Biology, School of Life Sciences, Xiamen University, Xiamen 361100, Fujian, China.

^*^ These authers contributed equally to this work

^#^Address correspondence to:

Jie Jiang, Department of Thoracic Surgery, The First Affiliated Hospital of Xiamen University, 55 Zhenhai Road, Xiamen 361003, Fujian, China; Tel: +86-(592)-2137270; E-mail: jjiang59@xmu.edu.cn

Qi-Cong Luo，Laboratory of Xiamen Cancer Hospital, The First Affiliated Hospital of Xiamen University, 55 Zhenhai Road, Xiamen 361003, Fujian, China; Tel: +86-(592)-2139518; E-mail: qcluo@xmu.edu.cn

Bo-An Li: Department of Biomedical Sciences, Xiamen University School of Life Sciences, D212 Huang Chaoyang Hall, Xiang’an Campus of Xiamen University, Xiamen, 361100, Fujian, China

Tel: 86-592-2181987; E-mail: [bali@xmu.edu.cn](mailto:bali@xmu.edu.cn)

Notes
The authors declare no competing financial interests.

Figure S1. The protein expression of all critical enzymes involved in glucose metabolism in A549 and SK-MES-1 cells overexpressing OVOL2.

Figure S2. P65 promotes the membrane translocation of endogenous GLUT1.

Figure S3. Nuclear P65 protein level is not regulated by OVOL2.


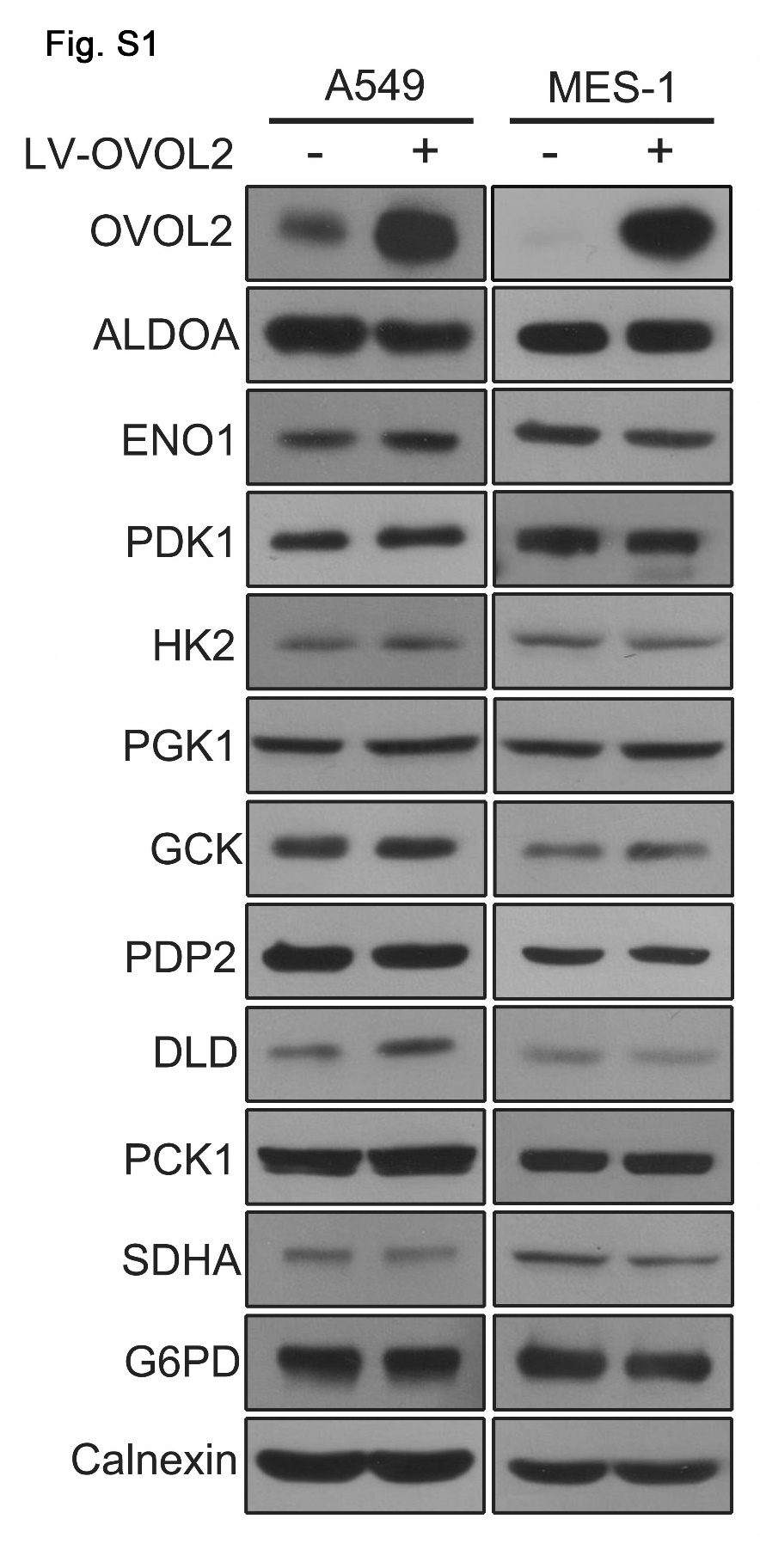


**Fig. S1. The protein expression of all critical enzymes involved in glucose metabolism in A549 and SK-MES-1 cells** **overexpressing OVOL2.** A549 and SK-MES-1 cells were infected with vector control or lentivirus-expressing OVOL2. All critical enzymes involved in glycolysis were measured by Western blotting.


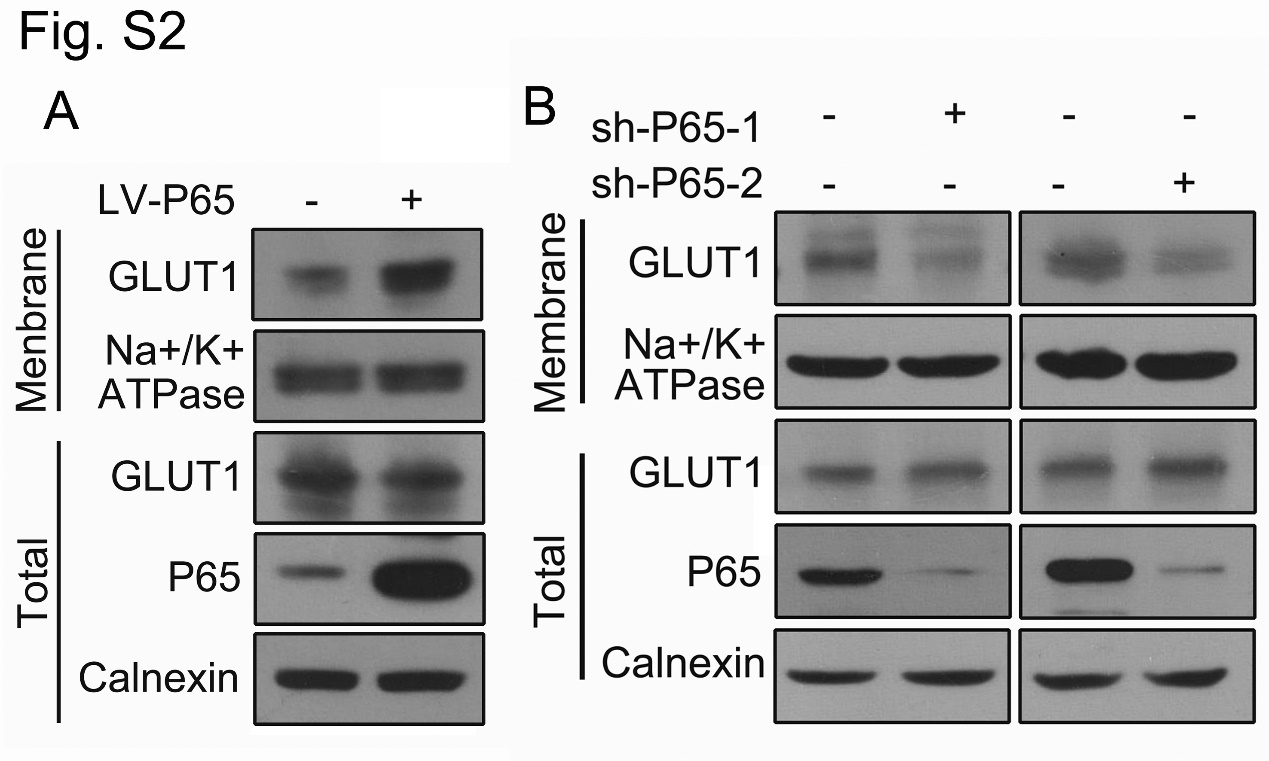


**Fig. S2. P65 promotes the membrane translocation of endogenous GLUT1.** P65 were overexpressed in NCI-H661 cells(A) or knocked-down in SK-MES-1 cells(B). Membrane GLUT1 expression were measured by Western blotting.


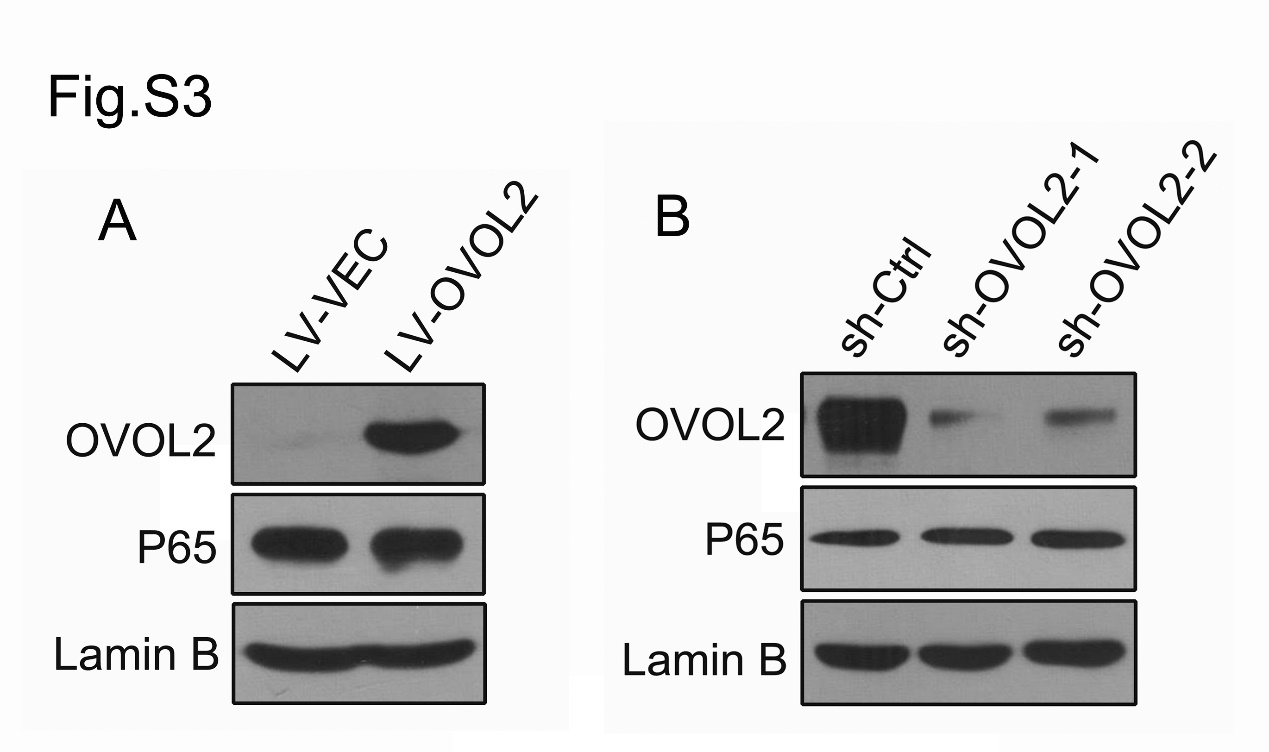


**Fig. S3.** **Nuclear P65 protein level is not regulated by OVOL2.** OVOL2 were overexpressed in SK-MES-1 cells (A) or knocked-down in NCI-H661 cells (B). Nuclear P65 protein expression were measured by Western blotting.
